# Variance Analysis in China’s Coal Mine Accident Studies Based on Data Mining

**DOI:** 10.3390/ijerph192416582

**Published:** 2022-12-09

**Authors:** Tianmo Zhou, Yunqiang Zhu, Kai Sun, Jialin Chen, Shu Wang, Huazhong Zhu, Xiaoshuang Wang

**Affiliations:** 1 Institute of Geographic Sciences and Natural Resources Research, Beijing 100101, China; 2 University of Chinese Academy of Sciences, Beijing 100049, China; 3 Information Institute of the Ministry of Emergency Management of PRC (IIEM), China Coal Information Institute (CCII), Beijing 100029, China; 4 Jiangsu Center for Collaborative Innovation in Geographical Information Resource Development and Application, Nanjing 210023, China; 5 Beijing Municipal Ecology Environment Bureau Integrating Business Center, Beijing 100048, China

**Keywords:** China, coal mine accidents, Apriori, LSI, variance analysis, data mining, CiteSpace

## Abstract

The risk of coal mine accidents rises significantly with mining depth, making it urgent for accident prevention to be supported by both scientific analysis and advanced technologies. Hence, a comprehensive grasp of the research progress and differences in hotspots of coal mine accidents in China serves as a guide to find the shortcomings of studies in the field, promote the effectiveness of coal mine disaster management, and enhance the prevention and control ability of coal mine accidents. This paper analyzes Chinese and foreign literature based on data mining algorithms (LSI + Apriori), and the findings indicate that: (1) 99% of the available achievements are published in Chinese or English-language journals, with the research history conforming to the stage of Chinese coal industry development, which is characterized by “statistical description, risk evaluation, mechanism research, and intelligent reasoning”. (2) Chinese authors are the primary contributors that lead and contribute to the continued development of coal mine accident research in China globally. Over 81% of the authors and over 60% of the new authors annually are from China. (3) The emphasis of the Chinese and English studies is different. Specifically, the Chinese studies focus on the analysis of accident patterns and causes at the macroscale, while the English studies concentrate on the occupational injuries of miners at the small-scale and the mechanism of typical coal mine disasters (gas and coal spontaneous combustion). (4) The research process in Chinese is generally later than that in English due to the joint influence of the target audience, industrial policy, and scientific research evaluation system. After 2018, the Chinese studies focus significantly on AI technology in deep mining regarding accident rules, regional variation analysis, risk monitoring and early warning, as well as knowledge intelligence services, while the hotspots of English studies remain unchanged. Furthermore, both Chinese and English studies around 2019 focus on “public opinion”, with Chinese ones focusing on serving the government to guide the correct direction of public opinion while English studies focus on critical research of news authenticity and China’s safety strategy.

## 1. Introduction

The complexity and hazards of coal mine disasters increase significantly with the depth of mining, triggering accidents of a serious nature at the slightest misstep. Following nearly 20 years of adjustment, the production safety level of China’s coal industry has made milestone progress—fatalities have dropped from 6458 to 191, and the coal mine fatality rate per million tons has dropped to less than 0.1 since 2018. Yet, unlike foreign countries where surface mines are widespread and highly mechanized, 81.37% of China’s coal mine capacity originates from underground mines. These coal mines are characterized by a wide range of distribution, highly varying occurrence conditions, as well as numerous and complex categories of coal mine disasters, which urgently need to be addressed by scientific and technological means to reduce the probability of coal mine accidents.

Given that the characteristics of coal mine disasters, accident prevention and control, and safety strategies vary significantly across different regions in China, accurately grasping the priority and potential hotspot issues of coal mine accident prevention and control is an essential prerequisite for increasing the efficiency of scientific and technological support, which also have received more and more attention of scholars. Numerous studies have conducted thorough analysis of coal mine accidents in China, achieving abundant achievements from appearance to mechanism: by 30 September 2022, the Web of Science (WOS) database contained about 13,700 papers on the topic of coal mine accidents or disasters in China (Topics are “China coal mine accident” or “China coal accident” or “China mine accident” or “China mine disaster” or “coal disaster” or “China coal mine disaster” or “China coal hazard” or “China mine hazard” or “coal mine hazard”), and the China National Knowledge Infrastructure (abbreviated as “CNKI” (CNKI refers to the China National Knowledge Infrastructure, which has collected more than 95% of officially published Chinese academic resources. Among them, there are more than 8490 academic journals, with a literature coverage rate of 99.9%. Foreign language academic journals covering 96% of JCR journals and 90% of Scopus journals. For this article, we had de-duplicated.), the world’s largest and most authoritative Chinese literature database [1]) contained about 12,900 papers on the same topic (topics are“煤矿事故” or “矿难” or “煤炭事故” (different forms of expressions for coal mine accidents in Chinese) or “煤矿灾害” (coal hazard)). Existing studies can be broadly divided into two categories: The first category is to conduct accident characteristic analysis from the perspective of accident type, high-incidence time, and high-incidence place to provide support for macroscopic grasp of China’s coal mine accident pattern in a specified time period, such as Chen [2], Deng [3], and Cui [4] using long time series coal mine accident data (generally more than 10 years of all-hazard accident information). Some of the studies only focused on the analysis of a single accident type, mainly for gas and water accidents. Some scholars, such as Dong [5], Li [6], and Yin [7], have conducted comprehensive studies on the environment that breeds disasters in coal mines, the spatial and temporal characteristics of accidents, and the major disaster-causing mechanisms, and have proposed basic countermeasures and recommendations. Other studies have been conducted on short-term coal mine accidents for roofs [8], electromechanical transportation [9], and fire [10]. The second category intended to analyze coal mine accident causation using a computational method. Scholars have introduced models and algorithms from other safety fields via direct use or algorithm improvement to conduct in-depth research on the causation of coal mine accidents. The highly cited literature in recent years has mainly concentrated on the application of traditional accident causation models such as Heinrich [11], Fault tree [12], and emerging models such as the 2-4 model [13,14] and HFACS [15]. The construction of accident causation chains to achieve the analysis of both direct and indirect causes of accidents is also a widely used model for the study of coal mine accidents in China.

Nevertheless, the abovementioned studies basically only analyzed the accident characteristics within the specified time period, with fewer conclusions of previous studies and comparative summaries, which led to a high similarity in the conclusions of many studies. That is, it is generally agreed that roof, gas, and transportation are the main accident types; township coal mines are the critical objects for accident management; the southwest and northeast regions are accident-prone areas, with Guizhou, Sichuan, Shanxi, Henan, Heilongjiang, Chongqing, and Yunnan as the key provinces; from October to the next January, from July to August, and from March to April are the accident-prone time periods each year in sequence; from 22:00 to 24:00 is the high-incidence period of coal mine accidents.

To better obtain the characteristics of studies in the field, reduce the occurrence of duplicate studies, and accelerate the study process, several scholars have analyzed the quantitative characteristics, publishing journals, research teams, and research topics of China’s coal mine accident studies in different time periods (1949–2014 [16], 1992–2018 [17], 2007–2016 [18], and 2000–2020 [19]). It is generally accepted that the study of coal mine accidents in China has phased characteristics, with the peak period of publications basically concentrated between 2005 and 2013; the year around 2008 marked a significant change in the research methodology; the research topics were focused on unsafe behavior, accident analysis, and safety management; the causation of accidents was mainly divided into human factors, system and law deficiencies, and supervisory oversight; and the application of computer technology increased significantly. Then, in combination with the previous search results, it can be observed that English-language journals were also the major publishing platform for China’s coal mine accident research, which has an important role in promoting the progress of studies in the field. Currently, the phenomenon of neglecting non-Chinese research findings in coal mine accident overview studies prevails in Chinese literatures, leading to the issue of being unable to fully reflect the research progress and hotspots in the field, thus affecting the guidance value of theoretical studies for practical work.

Hence, this paper adopts the data mining algorithms LSI and Apriori to explore the text of the literature published in Chinese and English journals for discovering the research patterns and development trends of China’s coal mine accidents, as well as to analyze the differences between Chinese and English studies. The findings are of practical significance for systematically and comprehensively grasping the research context of coal mine accidents in China and improving the accuracy and effectiveness of research directions as well as the selection of scientific and technological methods.

This paper is structured as follows. Section 2 introduces the rationale for the selection of the study time period, data sources, research methods, and data preprocessing processes. Section 3 analyzes the general variations in the volume and phased characteristics of the literature on coal mine accidents in China, as well as the characteristics of the variations in the Chinese and English literature. Section 4 outlines the phased topics of the Chinese coal mine accident research and describes the core topics of the Chinese and English studies and the strong association rules of keywords within the topics. Section 5 presents the potential hotspots of Chinese and English studies. Section 6 provides a discussion of the possible reasons for the discrepancies between Chinese and English studies.

## 2. Data and Methods

### 2.1. Data Selection and Preprocessing

#### 2.1.1. Data Selection

**Figure 1:**
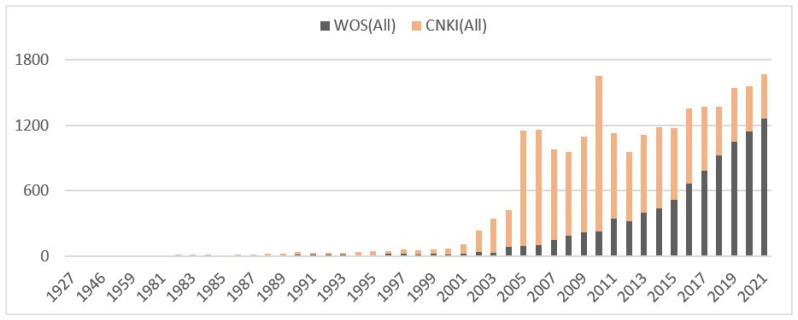
Number of studies on coal mine accidents in China based on preliminary statistics.

(1) Study time selection: China’s coal industry changed its industrial model from a direct state-investment to an autonomous operation in 1993 [20]. Correspondingly, the subversive change in the industrial model led to a shift from a decrease to an increase in the fatalities of coal mine accidents (China Economic and Social Data Platform in CNKI, name of yearbook is China Coal Industry Yearbook 2008, index is the number of fatalities of coal mine accidents (by accident type) nationwide over the years, https://data.cnki.net/HomeNew/index, accessed on 23 October 2022), which has attracted much attention from scholars. At the same time, the degree of industry data sharing has improved. It can be said that the actual problems have attracted the attention of scholars, and the research has the necessary data conditions. Moreover, the government-oriented operation before 1993 and an unwillingness for full disclosure prior to this date emerged. Therefore, the number of studies on coal mine accidents increased rapidly after 1993. The number of papers on China’s coal mine accidents that were included in the full WOS database or the full CNKI database before 1993 was less than 25 articles/year. However, the total number of papers on this topic has exceeded 50 articles/year since 1994 and has continued to increase (Figure 1). This indicates that the restructuring of China’s coal industry model has a driving effect on coal mine accident studies. For this reason, 1993 was chosen as the starting time for this study.

(2) Results type selection: As the papers on coal mine accidents in China are mainly published in Chinese and English-language journals, they can fully cover coal mine accident research, so only the results of these two languages were considered in this study. For English papers, WOS is the best and most popular literature database; especially, the Core Collection database in WOS is the world’s premier searchable database of core journals. To ensure the forward-looking and rigorous nature of the selected literature, only those papers included in this database are selected for the English literature. The Chinese studies are selected from CNKI, which consists of several subdatabases. In view of the significant advantages of the core journal database (equivalent to the WOS Core Collection) and the dissertation database (CNKI Dissertation Database: https://kns.cnki.net/kns8?dbcode=CDMD, accessed on 23 October 2022) (“Chinese Doctoral Dissertations Full-text Database” and “Chinese Master’s Theses Full-text Databases”) in terms of research hotspots identification and the scientific nature and academic influence of research results, the core journal papers and dissertations were selected.

#### 2.1.2. Data Preprocessing

**Figure 2:**
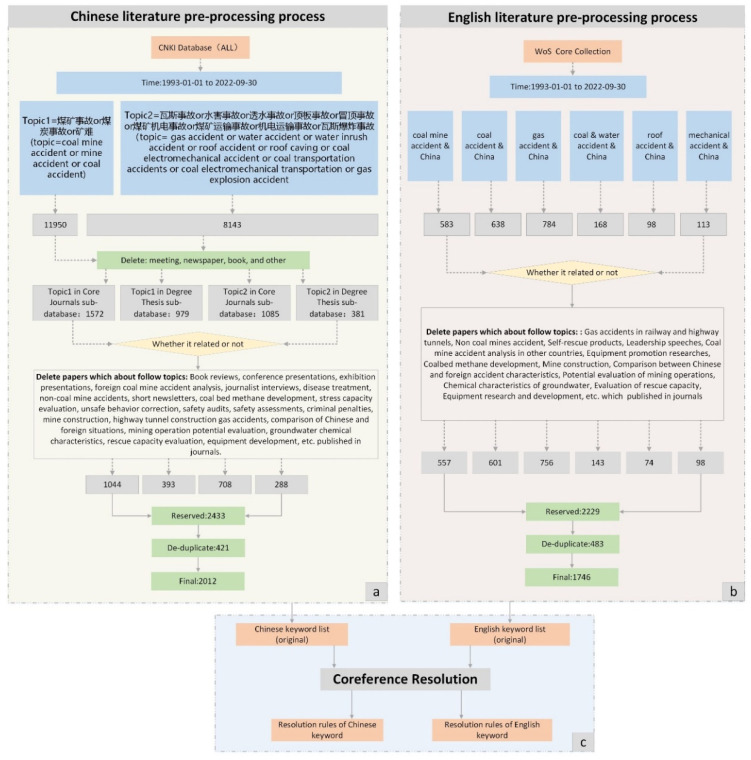
Data acquisition and preprocessing. (**a**) is Chinese literature pre-processing process; (**b**) is English literature pre-processing process; (**c**) is coreference resolution).

The data acquisition and preprocessing process is shown in Figure 2.

(1) Chinese literature: To enhance the coverage of retrieval, the search was conducted by the terms “煤矿事故 (coal mine accident)”, “煤炭事故 (coal accident)”, “矿难 (mine accident)”, and so on, and the retrieval was supplemented by the type of accident (“瓦斯事故 (gas accident)”, “水害事故 (water accident)”, “透水事故 (water inrush accident)”, “顶板事故 (roof accident)”, “冒顶事故 (roof caving accident)”, “煤矿机电事故 (coal mine electromechanical accidents)”, “煤矿运输事故 (coal mine transportation accidents)”, and “机电运输事故 (coal electromechanical transportation accident)”. The retrieval period was from 1 January 1993 to 30 September 2022, and the scope was core journal databases and dissertation databases, with a total of 4222 study records obtained. On this basis, the relevance of the literature topics was manually interpreted (the criteria are shown in Figure 2a), and then the literature was de-duplicated, invalid records were eliminated with the assistance of CiteSpace 6.1.R3, and, finally, 2012 Chinese records were retained.

(2) English literature: The topic was set as “coal mine accident & China” or “coal accident & China” or “gas accident & China” or “coal & water accident & China” or “roof accident & China” or “electromechanical accident & China” or “transportation accident & China”. The retrieval period was set from 1 January 1993 to 30 September 2022, and a total of 2384 study records were retrieved. After the manual interpretation and literature de-duplication (the criteria are shown in Figure 2b), 1764 records in English were finally retained.

(3) Coreference resolution: Coreference resolution refers to one of the major issues in natural language processing, which is mainly applied to identify and eliminate different representations referring to the same entity in the text and enhance the correct rate of text processing. This study adopted the manual rule definition method to perform coreference resolution on the corpus (Figure 2c). After obtaining the full list of Chinese and English keywords, the coreference phenomenon was identified, and then 392 rules were defined for both Chinese and English. For example: “behavioral safety 2-4 model”, “accident causation 2-4 model”, “24 model”, “2-4 model”, etc., all referred to the same term, i.e., the “‘2-4′ model”; “coal dust and gas explosion accident”, “coal dust explosion”, “coal dust explosion accident”, and “gas and coal dust explosion accident” all referred to “gas and coal dust explosion”; “coal mine supervision bureau”, “coal mine safety supervision bureau”, “state coal mine supervision bureau”, “mine safety supervision bureau”, “mine bureau”, and “state bureau” all referred to China’s coal mine supervision department.

### 2.2. Research Methods

This paper employs the Latent Semantic Indexing (LSI) model to uncover research themes and trends over time, which can provide a reference for clarifying the research context and revealing topical and cutting-edge issues in the field of coal mine accident studies. The implementation and visualization of the research methodology was achieved through CiteSpace (Official website of CiteSpace: https://sourceforge.net/projects/citespace/, accessed on 20 October 2022), a software commonly used in bibliometrics. Subsequently, the detection of strongly associated combinations in keywords was performed based on the Apriori algorithm, which provides support for identifying common combinations of keywords under each category of topics, as implemented through Python 3.10.

#### 2.2.1. Latent Semantic Index

Keywords are the core information reflecting the major research content of a paper. Thus, clustering analysis of keywords can contribute to discovering the inner connection of knowledge and revealing the trend of hotspots. Latent Semantic Indexing (LSI) is an automatic knowledge extraction and representation method that is widely applied in information retrieval applications such as natural language understanding and text classification. The method assumes that there is a close association among terms and produces mapping rules between terms and semantics by extracting the contextual meaning of the terms after statistical analysis of the text [21]. This paper intends to use the LSI algorithm to cluster keywords of Chinese and English coal mine accident studies separately so as to obtain the main categories of Chinese and English research topics.

The LSI method maps the high-dimensional vector space H to the low-dimensional latent semantic space (LSS) by singular value decomposition (SVD) [22]. The co-occurrence is represented by constructing the *m* × *n* dimensional feature matrix and then decomposing H into the product of three matrices [23] (as shown in Equation (1)), taking the first l columns $S_{l},\;D_{l}$ of *S* and *D* to form the approximation matrix $H_{l}$ of *H* (as shown in Equation (2)), and the LSI model is achieved by CiteSpace software in this study.

(1) $$H={\left[h_{ij}\right]}_{m\times n}=S_{m\times n}V_{m\times n}D_{n\times n}^{T}$$

(2) $$H\approx H_{l}=S_{l}V_{l}D_{l}^{T}$$

where H is the high-dimensional space, $h_{ij}$ is the weight of the ith term in the jth text, m is the total number of feature words in the text set, and n is the number of texts. $S_{m\times n}$ and $D_{n\times n}^{T}$ are the left/right singularity matrices of *H* (both orthogonal), respectively, with the row vectors in the former matrix corresponding to the word vectors of the original matrix *H* and the row vectors in the latter corresponding to the document vectors of the original matrix *H* [24]. $V_{m\times n}$ is a diagonal matrix, which is a sequence of singular values of H ($\alpha_{1},\;\alpha_{2}\ldots \alpha_{k},\;\mathrm{and}\;\alpha_{1}\geq \alpha_{2}\geq \ldots \geq \alpha_{k}>0$).

#### 2.2.2. Apriori Algorithm

Several topic clusters and the set of terms composing the clusters can be obtained by the LSI method, yet the high frequency combinations within the set and the degree of association among terms cannot be clarified. Hence, the strongly associated combinations of Chinese and English keywords are explored by the Apriori algorithm, respectively. The algorithm is widely used as a classical association rule mining algorithm in numerous fields [25,26,27] and provides support for detecting potential relationships among multiple research objects. By employing this algorithm, the Chinese and English keywords were calculated separately to further reveal the common keyword combinations for different research topics.

The Apriori algorithm is used to discover itemset relationships from massive data by searching layer by layer and forming strongly associated rules that match the filtering criteria [28], that is, discovering frequent itemset K by scanning the data and noting it as k1, searching for frequent itemset K + 1 based on it and noting it as k2, and so on, till no new itemset is found. For discovering and determining strongly associated rules, the degree of support and confidence of the frequent itemset cannot be lower than user-defined thresholds, and only rules that satisfy both can be selected. This study was implemented via Python.

#### 2.2.3. CiteSpace Software

The CiteSpace software is a literature visualization analysis software developed by Chaomei Chen’s team against the background of scientometrics and data visualization and is the common technical method in the current bibliometric field. The present study realized literature preprocessing, the visualization presentation of LSI algorithms, and research results via this software.

## 3. Overall Trends of China’s Coal Mine Accident Studies

### 3.1. Quantitative Characteristics of the Literature

**Figure 3:**
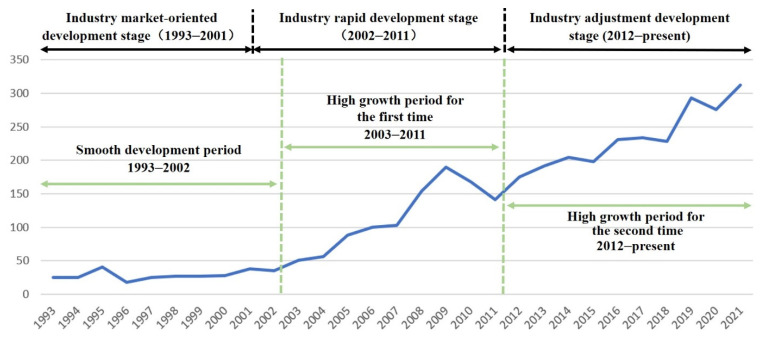
Overall trends in the number of papers on coal mine accidents in China.

The continued growth in the number of papers on China’s coal mine accidents during the study period (as shown in Figure 3) indicates that the topic continues to gain influence internationally and remains one of the topical issues in the field of coal mine safety.

Taking into account the development history of China’s coal industry [20], it can be observed that the research history of coal mine accidents in China basically conforms to the characteristics of industrial development stages; that is, 2002 and 2012 are the critical node years. ➀ Before 2002, the research in this field was at the exploration stage, and the number of papers was less than 50/year, with the research content mainly focusing on the description of the accident and the treatment results. ➁ The number of papers started to grow from 2003 to 2011. During this period, there was a high incidence of coal mine accidents in China, and the concentrated outbreak of various types of potential risks aroused intense attention from scholars worldwide. ➂ From 2012 onwards, the research entered the second rapid phase, with the number of papers being twice as much as the previous phase and maintaining this growth trend, indicating that coal mine accidents in China continue to be one of the major areas of concern for scholars.

### 3.2. Author Distributions

**Figure 4:**
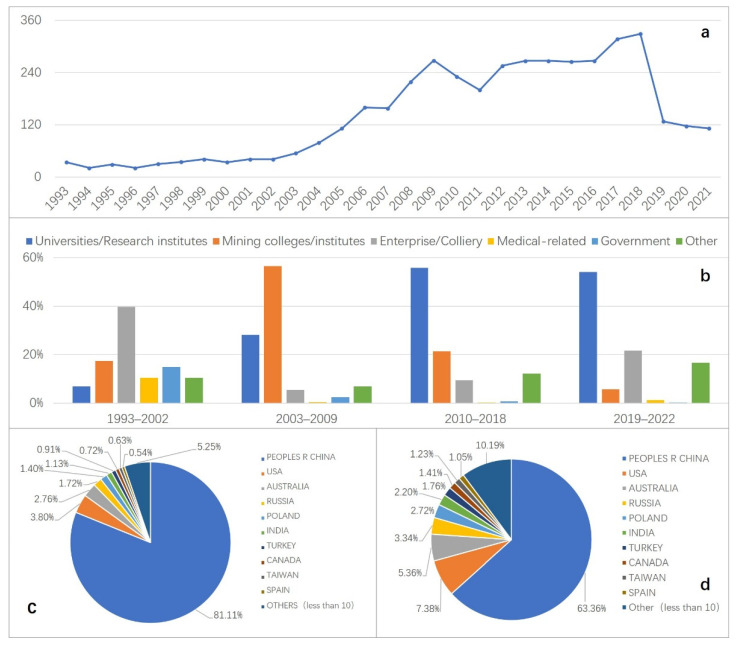
Author distributions (**a**) number of new authors per year, (**b**) distribution of new authors by institution, (**c**) distribution of all authors for both Chinese and English-language papers, (**d**) distribution of authors by nationality for English papers).

The total number of authors of studies on the topic of coal mine accidents in China during the period of 1993–2022 is 4523. By combining the changes in the number of new authors (Figure 4a) with the institutions of authors (Figure 4b), it can be observed that the change in the number of new scholars can be divided into four phases: stable development period (1993–2002), high growth period (2003–2009), fluctuating growth period (2010–2018), and uniform decline period (2019–2022), and the change of author type from mainly employees of enterprises to mainly researchers (universities/research institutes, mining colleges/institutes) indicates that the issue of coal mine accidents in China is increasingly attracting more and more academic attention. It should be noted that about 81% of all authors are from China (Figure 4c), with more than 60% of new authors from China annually. Furthermore, more than 63% of the authors of the English literature are from China (Figure 4d), and the top 100 authors who have published papers in English in aggregate (36% of all English publications) are all from China. This indicates that Chinese authors are the primary contributors of the continued development of China’s coal mine accident research.

### 3.3. Categories of Research Topics

**Figure 5:**
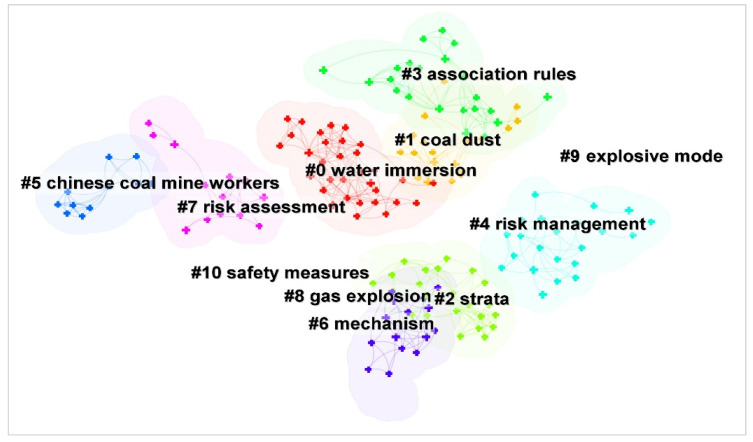
Categories of topics for research on coal mine accidents in China.

Based on the keyword clustering outcomes (as shown in Figure 5), research on coal mine accidents in China is mainly divided into 11 topics, which can be further combined into four major categories.

The first category is statistical analysis (#0, #1, #8), which is a topic throughout the research period. It mainly analyzes the characteristics and changes of China’s coal mine accidents in the specified area or time through statistical or operational methods, with gas explosion, coal dust, water damage, and roofs being the major objects of study. The second category is causation analysis (#2, #3, #5, #6, #9), which is a study of accident patterns and mechanisms such as influence factor analysis and association rule extraction to support the reduction of the number of accidents and fatalities. The third category is risk management (#4, #7), which mainly includes hazard source identification, risk index management, accident risk evaluation, and so on. The fourth category is safety management (#10), involving government-enterprise game, safety supervision, emergency rescue, safety culture construction, mechanism and system, public opinion analysis, technology update, and other studies based on coal mine accident prevention and control, and combined with multiple fields, which is a potential collection of hotspots for coal mine accident studies.

## 4. Analysis of Differences in China’s Coal Mine Accident Studies

### 4.1. Difference in the Number of Chinese and English Studies

**Figure 6:**
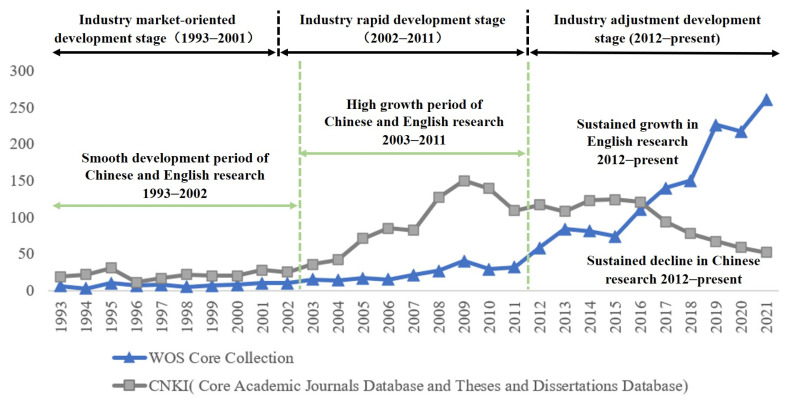
Number of Chinese and English papers (screened).

The variation characteristics of the number of Chinese and English papers on coal mine accidents in China differ greatly (Figure 6). In particular, the number of Chinese papers continued to increase from 1993 to 2009; the research output decreased from 2010 to 2016, with fluctuating changes around 120/year. From 2017 onwards, the number of papers published continued to decrease at a rate of 15%/year. The number of English studies was relatively low before 2011 (with an average number of 15/year), the number of results increased significantly from 2012 to 2016 (with an average number of 80/year), and the number of English papers published exceeded the Chinese papers from 2017 and maintained a rapid growth trend. The reasons for this phenomenon may be related to the sharp decrease in the sample size of coal mine accidents in China, changes in research topics, and changes in authors’ intention to publish their results, which need to be analyzed thoroughly.

### 4.2. Differences between the Authors of Chinese and English Studies

**Figure 7:**
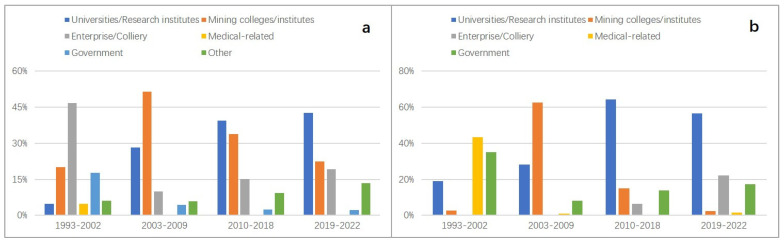
Distribution of new authors by institution (**a**) for studies in Chinese; (**b**) for studies in English).

As shown in Figure 7, from 1993 to 2009, the trend of changes in the number of authors for both Chinese and English studies were the same. In particular, the increase in the number of new scholars per year from 1993 to 2002 was stable, with an average of 5 new authors per year in English and about 30 in Chinese, which indicates that the status of both Chinese and English studies and the source of new scholars were relatively stable during this period. The average annual growth rates of new authors in Chinese and English from 2003 to 2009 are with 29.73% and 32.86%, respectively. As the safety situation in China’s coal mines deteriorated and the level of data sharing increased, coal mine accident research began to attract considerable academic attention, and the type of authors changed from “enterprise employees” (Chinese) and “medical workers” (English) to “mining colleges/institutes” and “universities/research institutes”. Participation of scientific researchers is also one of the major reasons for the surge in the number of papers at this stage.

The number of new scholars fluctuated from 2010 to 2018, with the total number basically remaining at 280/year, yet the characteristics of the change in the number of new authors for English and Chinese papers were diametrically opposed. More than 56% of the new authors of English studies in this period came from Chinese research fields (universities/research institutes/mining colleges/institutes). It is possible that some scholars entered into the field of coal mine accident research by publishing their papers in Chinese and then switched their publication methods and submitted their new findings to the English journals, taking into account the prevalence of “SCI determinism” since 2011 [29,30]. Consequently, the trend of new authors in Chinese and English is opposite in this phase.

The number of new authors in English studies showed a precipitous decline from 2019 to 2021, while the number of new authors in Chinese turned from a decline to an increase, with more than 65% coming from the scientific research field. This could be directly related to the announcement of “eliminating the talent evaluation system only based on papers, titles, degrees and awards” (On 23 October 2018, the Ministry of Science and Technology, the Ministry of Education, the Ministry of Human Resources and Social Security, the Chinese Academy of Sciences and the Academy of Engineering jointly issued the “Notice on the Special Action to Eliminate the Talent Evaluation System only based on ‘Papers, Titles, Degrees and Awards’” (Government Document (2018) No. 210 issued by the Ministry of National Science and Technology) referred to as “Eliminate the Four Onlys”. The purpose of this special action is: there are still unreasonable phenomena in the talent evaluation system, which urgently needs to emphasize the orientation of moral character, competence, and performance, to overcome the tendency of evaluating talents by papers, titles, degrees, and awards, and to implement the representative work evaluation system, focusing on the quality, contribution, and influence of emblematic achievements. https://www.most.gov.cn/xxgk/xinxifenlei/fdzdgknr/fgzc/gfxwj/gfxwj2018/201902/t20190213_145084.html, accessed on 23 October 2018) in China at the end of 2018, and the change of the research evaluation system has pushed Chinese authors back to Chinese journals.

### 4.3. Differences between Chinese and English Research Stages

Taking into account the important years (1997, 2002, 2012, 2017, and 2020) (the outbreak of the Asian financial crisis in 1997 brought a huge impact on China’s coal industry, which had just entered the market-oriented development stage, bringing industrial development to a standstill, with a serious lack of safety investment and a deteriorating safety situation. The decade of 2002–2011 was the golden decade for China’s coal mines. The years of 2012–2016 are known as the chilly four years. The year 2020 marked the launch of the “carbon neutrality and carbon peak” objective.) and the number of papers (2002 and 2011) about China’s coal industry, the keywords were counted in stages according to six time periods: 1993–1996, 1997–2001, 2002–2011, 2012–2016, 2017–2019, and 2020–2022. The top 10 words in each stage were selected as the core words in that stage. It should be clarified that, in light of the large time span from 2002 to 2011, the top 20 keywords were selected as the core words. When selecting high-frequency words, “China”, “coal mine”, “accident”, and “coal mine accident” were no longer considered.

**Figure 8:**
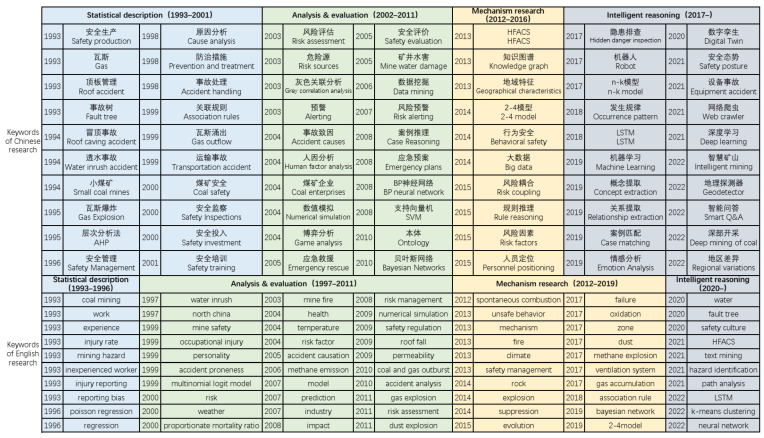
The evolution of keywords in English and Chinese studies.

According to the statistical results (Figure 8), it can be observed that China’s coal mine accident studies basically follow four stages: statistical description, analysis and evaluation, mechanism research, and intelligent reasoning. Nevertheless, the time periods corresponding to the Chinese and English studies are slightly different, with the Chinese studies following the four time periods of 1993–2001, 2002–2011, 2012–2016, and 2017–2022, and the English studies following the four time periods of 1993–1996, 1997–2011, 2012–2019, and 2020–2022. The characteristics of the different stages of the study are:

(1) Statistical description stage: After China’s coal industry entered the market-oriented development period [31], the safety production issues became increasingly prominent (coal mine fatality rate per million tons increased from 4.65 to 5.71). At this point, both domestic and international studies on coal mine accidents in China were in their initial stages, with relatively little research content and few methods. Domestic studies (1993–2001) were mainly based on statistics, fault trees, hierarchical analysis, and gray correlation analysis to provide a brief statistical description of a single accident or coal mine accidents in a specified region (nationwide/specified provinces). The international studies (1993–1996) mainly employed linear regression methods to analyze the situation around the injuries caused by coal mine accidents to miners. Studies at this stage were generally characterized by a single indicator system and low reusability, with a preference for the analysis of a single event. 

(2) Analysis and evaluation stage: The period from 2002 to 2011 was the “golden decade” of China’s coal industry [32], which was also the period with the most severe situation of coal mine safety production and high incidence of major and extraordinary accidents. Chinese studies started to focus on the evaluation of unsafe behaviors, risk assessment, hazard source identification, and emergency rescue based on the description of accident characteristics and introduced accident models from other safety fields to try to analyze the causal factors of coal mine accidents. Moreover, there were also papers focusing on the evaluation of regulatory and supervisory agencies with the keywords of “collusion between government and coal mining enterprises, safety supervision, and coal supervision bureau”. Such papers, however, have more obvious characteristics of the times. After the establishment of a national system of coal mine safety infrastructure in 2008 [33], studies on the government-enterprise game decreased significantly. The English studies entered the risk evaluation stage earlier (1997–2011), and the objects of the studies were still mainly miners. One category is to further improve the risk evaluation index system and quantify the impact of coal mine accidents on miners’ life and health through the identification of subjective factors (health status, personal character, stress resistance, and so on) and the analysis of the impact of objective factors (occurrence conditions, operating environment, industrial chain, and so on) on subjective factors. The other category is the study of safety production risk identification and assessment, further risk simulation, especially the study of gas transportation and permeability calculation, and mine pressure law.

(3) Mechanism research stage: With the sharp decline of coal prices, China’s coal industry entered the “chilly four years” [34], which was the period of deep adjustment of the industry. In this period, the studies focused on the investigation of the accident mechanism. Among them, the Chinese studies (2012–2016) were more interested in analyzing the human factors of national coal mine accidents (especially gas accidents) through improved algorithms (“2-4” model, HFACS, and so on) and gradually introduced text mining methods to identify and extract massive amounts of network texts. These studies provided support for exploring the degree of association among accident causation factors and potential rules, constructing accident causation chains and developing preventive measures in a targeted manner. The temporal and spatial scope of the English studies (2012–2019) is generally smaller than that of the Chinese studies, and mainly focused on a single coal mine, a single province, or a single accident category. According to the types and regions of coal mine disasters, studies were conducted from the perspectives of nurturing environment, human factors, policy orientation, and electromechanical equipment to quantify the severity and evolution patterns of production safety risks. English studies of accident mechanisms have a longer duration and more-detailed scale, with a tendency to analyze specific issues thoroughly.

(4) Intelligent reasoning stage: With the enactment and implementation of a series of industrial policies, the situation of coal mine safety production tends to be stabilized, and the focus of Chinese studies (2017–present) changed to risk monitoring and early warning, in-depth discovery of accident causal chains and potential rules, and text mining-based decision support. The possible risk points in the coal mining process were automatically discerned and matched with cases to achieve advance prevention of coal mine accidents. Furthermore, with the rapid advancement of deep mining, renewal of electromechanical equipment and intelligent construction, scholars started to concentrate on research related to coal mine electromechanical equipment and the regional variations of coal mine safety levels. English studies still focused on coal mine disaster mechanisms, with more papers based on machine learning methods for analysis of coal mine accident investigation reports appearing in 2020. Overall, however, the topics of the study were similar to the Chinese studies from 2017 to 2019.

### 4.4. Differences between Chinese and English Research Topics

#### 4.4.1. Chinese Research Topics

In this study, the LSI algorithm was implemented through CiteSpace to perform text clustering analysis of keywords, which yielded a total of 953 Chinese keywords and 2235 keywords links, with a Q value of 0.7125 and an S value of 0.896. This indicated that the clustering results were significant, the homogeneity of nodes within the same cluster was high, and the results were credible. The clustering results were further coalesced into three major categories based on the content, including accident statistical analysis, accident causation exploration, and coal mine safety management.

(1) Accident characteristics statistics: The statistics involved five topics: roof accidents, gas accidents, water accidents, fire accidents, and comprehensive analysis, covering six keyword clusters (as shown in Table 1). The keywords were highly homogeneous, and the peak years of topic clustering appeared around 2000 and 2010, respectively. Statistical [2,3,6] and operational [35,36,37] methods were mainly employed to quantitatively evaluate the information of coal mine accidents in a specified time period or specified spatial range, and then to summarize accident patterns and propose prevention and control measures. The results of the study provided a reference for grasping the characteristics of China’s coal mine accidents from a macro perspective.

(2) Exploration of accident causation (as shown in Table 2): The first peak of studies appeared around 2008. During this period, the fault tree analysis [10,38,39], hierarchical analysis [40,41,42], and gray correlation analysis [43,44,45] were mainly employed to carry out preliminary exploration on the causation factors of coal mine accidents in China. The second peak period was in 2011. During this period, with the increasing awareness of the view that “human causes are the primary contributors to production safety accidents” [46,47,48], the direction of studies was gradually divided into two categories: human factor analysis and comprehensive influence factor analysis. The former mainly focused on gas accidents and classified the causation of accidents and achieved the construction of causation chains for specific types of coal mine accidents through the “2-4” model [49,50,51] and the human factors analysis and classification system (HFACS) [52,53,54]. The latter mainly focused on the comprehensive analysis of both internal and external factors such as unsafe actions and unsafe physical states that cause accidents from the perspectives of coal mining conditions [55], equipment and facilities [56], technology level [57], mechanisms and systems [58], and safety management [59]. The third peak period was in 2021. During this period, texts were automatically extracted and graphically managed by introducing machine learning algorithms to significantly improve the processing efficiency of massive data. As an emerging method for in-depth discovery of knowledge in the field, it was mainly applied to the conceptual system construction and entity identification research for coal mine accident ontology construction [60,61,62,63], equipment information ontology database construction [64,65,66], monitoring and early warning [67,68,69,70], and case management [71,72,73,74].

(3) Coal mine safety management (as shown in Table 3): This covered the study directions of government-enterprise game, monitoring and early warning, safety culture construction, emergency rescue capacity building, and so on. The period around 2005 marked the rapid development of China’s coal mine industry. As a result, papers on enterprise safety culture building, illegal mining, and small coal mine rectification [75,76,77,78] appeared centrally in this period. Since then, the topics of papers have expanded to the construction of governmental regulatory models and mechanism systems [79,80,81] and the dynamic game between government and enterprises [82,83,84]. The topics of monitoring and early warning gradually emerged around 2012, and the identification of potential coal mine safety risks, disaster monitoring, and early warning became one of the major directions of coal mine accident studies. Among them, gas concentration monitoring simulation [85,86,87] and prediction of shock bump risk [88,89,90] were the core contents of the study. Around 2017, studies on information systems combined with GIS technology emerged around the requirements of coal mine accident disposal and emergency rescue. Such studies mainly focused on the systematic management of possible situations and solutions before and after an accident from the perspective of emergency planning and emergency disposal methods and provided query and retrieval services on demand [91,92,93].

(4) Strong association rules for Chinese keywords

**Figure 9:**
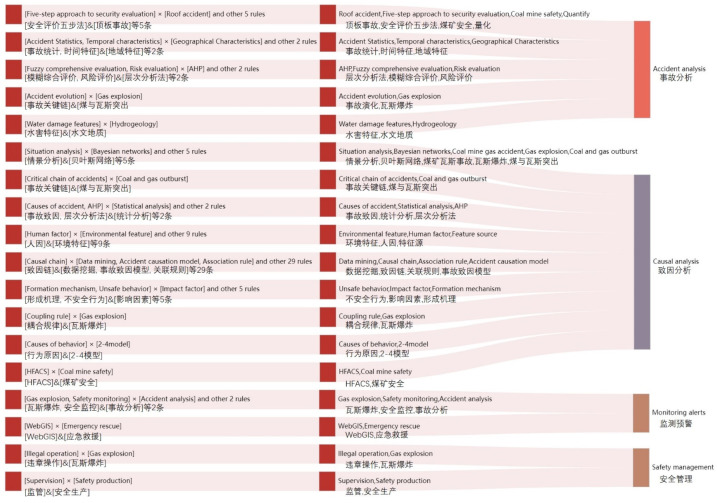
Strong association rules and category of Chinese keywords.

A total of 78 strong association rules with a confidence level greater than 0.8 were obtained based on the Apriori association algorithm. After grouping the 78 Chinese keyword rules according to their content attributes, it was found that they could be combined into four categories of 18 combinations of keywords, which were divided into causation analysis, statistical analysis, monitoring and early warning, and security management (as shown in Figure 9). In terms of the causation analysis, the strong association rules basically contained a class of algorithms or models. The strong association rules of accident analysis mainly reflected different perspectives of accident characteristics analysis, such as characteristics statistics, risk evaluation, safety evaluation, and so on. The strong association rules of monitoring and early warning category were mainly developed around the information system. The safety management category focused on the supervisory ability and operation against rules.

#### 4.4.2. English Research Topics

Statistics revealed that the total number of English keywords were 606, the total number of keyword links were 2323, the Q value was 0.7721, and the S value was 0.9076. The results indicated that the clustering was remarkable, and the homogeneity of nodes within the same cluster was high, with credible outcomes. As with the Chinese studies, the keywords of English studies were further combined into three major categories based on the topic directions involved in the clustering, which were: occupational injury analysis, disaster mechanism study, and coal mine safety management.

(1) Occupational injury analysis: Most of the international studies on coal mine accidents in China were focused on “people” and the injuries caused by accidents to miners (as shown in Table 4), which were divided into descriptive statistics and quantitative analysis. The former was based on behavioral data of the personnel involved, obtaining age, health status, behavioral characteristics, individual personality, experience level, working conditions, and environmental characteristics [94,95,96]. Studies on occupational injury category were inclined to the construction of safety and health index system, injury evaluation, and association analysis of influencing factors [97,98,99].

(2) Disaster mechanism study: The studies were mainly divided into seven topics, including gas explosion, water damage, coal and gas outburst, coal mine explosion, and fire (as shown in Table 5). Gas accidents were always the major study object [100,101], with fire and water accident types appearing more often in the first decade of the 21st century [102,103,104]. Studies on accidents in water, roof, and surface coal mines have gradually increased, which was associated with the increase in the frequency of extreme weather and the aggravation of compound disasters. During 2010–2020, international studies on coal mine accidents began to concentrate on comprehensive evaluation, correlation analysis among accidents and among their causation factors, safety risk evaluation and risk prediction by means of neural networks and correlation algorithms [105,106,107], as well as replication of accidents through laboratory simulation experiments [108,109,110].

(3) Coal mine safety management (Table 6): As shown by the previous studies [33], China’ coal mine safety production levels were in an unstable state from 2008 to 2015. As a result, the papers on corporate safety culture building [111,112,113], safety atmosphere (miners’ work–family atmosphere, socio-economic and safety atmosphere, and safety atmosphere at the enterprise leadership level) [114,115,116], coal mining capacity and risk assessment, and government-enterprise game and management effectiveness analysis [117,118,119] were centralized in this period. News coverage of coal mine accidents in China became a popular direction of attention for some scholars around 2019 [120,121,122], who assessed the social impact of accidents and the leading role of social opinion by analyzing the methods and approaches of news coverage.

(4) Strong association rules for English keywords

**Figure 10:**
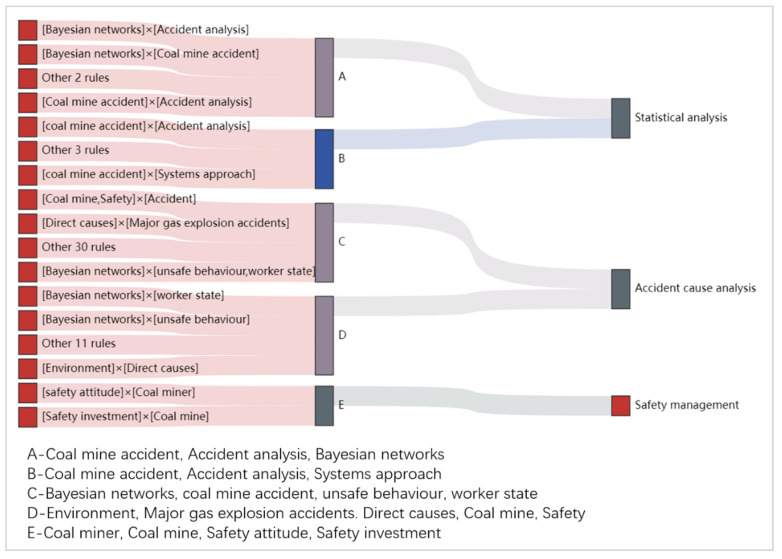
Strong association rules and category of English keywords.

A total of 59 strong association rules with a confidence level greater than 0.8 were obtained based on the Apriori association algorithm. These rules were mainly focused on statistical analysis, causation analysis, and safety management (as shown in Figure 10). In particular, Bayesian networks and systematic methods were commonly used for statistical analysis; the causation analysis was mainly focused on miners’ unsafe behaviors; safety management was more focused on safety inputs and miners’ safety attitudes.

### 4.5. Differences in the Trends between Chinese and English Studies

#### 4.5.1. Overall Differences in the Evolution of Hotspots

**Figure 11:**
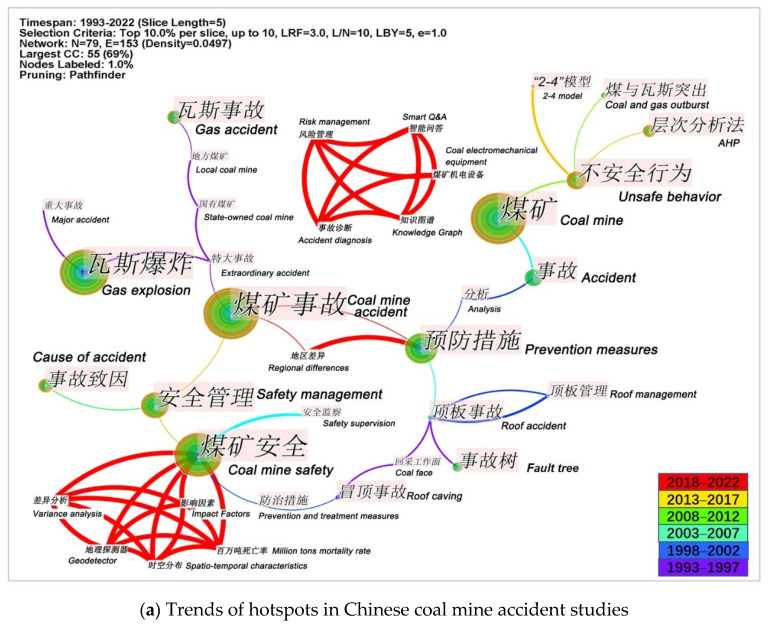

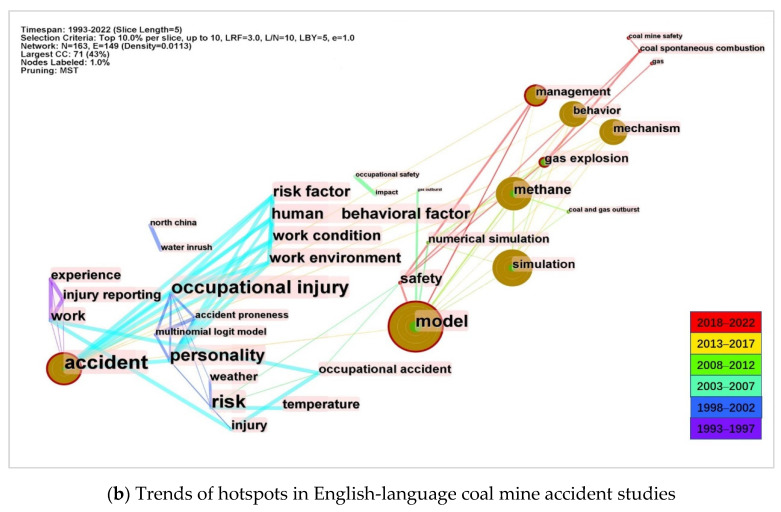
Trends of hotspots in Chinese and English coal mine accident studies.

There were two main directions of potential hotspots in China’s coal mine accident studies (Figure 11a). The first was the study of regional differences (spatial and temporal differences in accidents, geographical differences in causation, and so on), which mainly focused on the automatic mining of massive, multimodal data through machine learning algorithms. The other one was the unsafe behavior analysis, which combined text mining algorithm and “2-4” model to enhance the automatic extraction of accident causation in massive accident text. In contrast, the potential hotspots of English studies (Figure 11b) remained basically unchanged, mainly in the three areas of coal mine disaster mechanism, behavioral safety, and numerical simulation, and the study objects were still mainly coal and gas, and there were papers with coal spontaneous combustion as the study object. Furthermore, coal mine accidents were characterized by chains, and gas accidents were one of the accident categories that caused the greatest human and economic losses. Consequently, gas-related co-occurring disasters have gradually become a study hotspot mainly focused on the co-occurring sensitivity coefficients of gas and coal spontaneous combustion, gas, and water gushing-out, and the study of gas permeation rate category in large-mining-height mining areas.

#### 4.5.2. Hotspot Differences and Trends in the Last 5 Years

**Figure 12:**
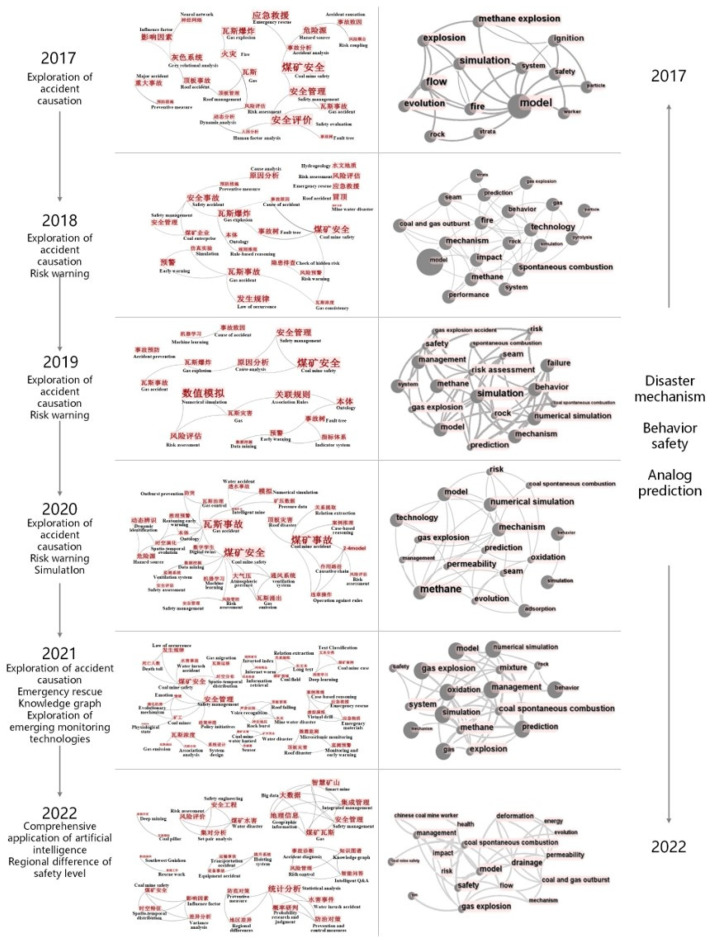
Comparison of Chinese and English research hotspots during 2017–2022.

To better identify the frontier hotspots of coal mine accident studies in China, the topics of studies were further analyzed based on the literature records from 2017–2022 (as shown in Figure 12). Topics of the English studies focused on three directions: disaster mechanism, behavioral safety, as well as simulation and prediction, with “gas/explosion”, “coal spontaneous combustion”, “numerical simulation”, “mechanism”, “prediction”, “miner/behavior”, and “risk” being the core terms for each year. Topics of the Chinese studies changed significantly, following the order of “causation analysis (2017), causation analysis & risk warning (2018, 2019), causation analysis & risk warning & simulation and prediction (2020), causation analysis & emergency rescue & knowledge graph application & exploration of emerging monitoring technologies (2021), integrated application of AI & regional differences in safety level (2022)”.

From the perspective of the study objects, Chinese studies have progressively focused more on water damage in coal mines, which is directly related to the frequent occurrence of extreme weather in China [33], while the English papers continuously focused on gas explosion and coal spontaneous combustion. From the perspective of technical methods, Chinese studies pay more attention to automatic reasoning of accident cases and application of AI technology. In particular, after the implementation of the “carbon neutrality and carbon peak” target in 2020, AI-based text mining, intelligent management, monitoring, and early warning research has rapidly become the main direction of Chinese studies. Through text mining algorithms and knowledge graph technology, scholars have been able to deeply reveal potential connections among coal mine disasters and to realize knowledge logic management, thus achieving intelligent support for knowledge in the field and automatic diagnosis of accident risks. During this period, topics of English studies were similar to those of the previous period, and the “2-4” model commonly used in Chinese studies began to appear frequently in behavioral safety analysis methods and attempted to carry out automatic parsing of accident investigation reports through text mining methods. Overall, the Chinese study topics in 2018–2020 moved rapidly closer to the English topics, and from 2021 onwards, the Chinese studies obviously focused on the application of AI technology in coal mining, while the English studies still focused on the exploration of disaster mechanisms.

As Internet technology develops, public opinion analysis becomes a vital part of all industries, and studies in English and Chinese around 2019 focused on articles related to “accident reporting”, “accident concealment”, or “news opinion”. Among them, the Chinese studies employed text clustering algorithms to discover the public opinion topics and public sentiment trends of coal mine accidents to provide support for the government to guide the correct and positive direction of public opinion [123,124,125]. The English studies, on the other hand, focused on accident concealment or how the news was described [120,126,127], and conducted a critical study of China’s coal mine safety strategy by evaluating the role assumed, the role played, and the impact generated by news organizations in accident reporting. It should be noted that the authors of the English public opinion studies were mainly from China, indicating that Chinese scholars began to confront the issue of safety production in China with a more liberal mind and dialectical thinking, with more attention to the origin and authenticity of the data. The emergence of critical studies further indicated that scholars are adhering to a more dialectical and rigorous attitude when facing scientific issues.

## 5. Discussion

It is evident from the analysis of the Chinese and English-language papers on coal mine accidents in China over the past 30 years that the research topics on coal mine accidents have varied with the changes in China’s coal industry policies. Nevertheless, prior to 2018, Chinese research topics were slightly behind the English studies in terms of their frontiers, and there were also differences in research. More specifically, the Chinese research mainly focused on “accidents”, while the English research mainly focused on “people”. These characteristics may be explained by the following reasons.

(1) Changes in policy orientation of industry and research evaluation led to differences in the progress of English and Chinese studies. Chinese scholars have a language advantage in obtaining and understanding China’s coal industry policies, allowing them to grasp the development direction more accurately. However, under the influence of China’s scientific research evaluation system, a great number of academic results flowed to the SCI journal [128]. After China launched the initiative of “eliminating the talent evaluation system solely based on papers, titles, degrees and awards” in 2018, scholars have become more willing to express their academic views in Chinese journals. In 2020, after the implementation of the “carbon neutrality and carbon peak” objective, the transformation of the coal industry model stimulated a rapid change in the direction of research in the field, coupled with the scholars’ freedom from concerns about the research evaluation system, a large number of excellent research results quickly returned to Chinese journals. This change pushed the Chinese research into a new development stage ahead of the English research, that is, from theoretical methods to higher-level early warning and forecasting research led by integrated application of AI. 

(2) The scope of research audiences affects the differences between Chinese and English research perspectives. Chinese research mainly serves to improve the national or regional coal mine safety production level and provides reference for governmental decision making. Therefore, scholars pay more attention to the overall characteristics and trends of regional or national coal mine accidents when expressing, in Chinese, to improve the dissemination efficiency and adoption probability of research results. The English papers, on the other hand, have relatively less impact on Chinese government officials due to language differences and accessibility. For this reason, the authors’ research is more in line with the international perspective when writing in a non-Chinese language; that is, they focus on the analysis of human behavior and the extent of damage to people caused by coal mine disasters.

Second, in comparison with previous studies, the present study further refines the developmental phases of coal mine accident studies in China, summarizes the thematic characteristics and major approaches at different phases, and explores the hotspot trends in both Chinese and English studies. In combination with the previous achievements, this study suggests the contents that still need attention for research in the field.

(1) Expand the research object. As coal mine disasters have chain characteristics, and the issues of self-coupling of coal mine disasters and coupling of natural disasters and coal mine disasters have increased significantly, more attention should be paid to the study of composite coal mine disasters, especially coal spontaneous combustion & gas, precipitation & water damage, earthquakes& roof safety. Apart from the impact of objective factors such as coal mine occurrence conditions and natural disasters, various subjective and objective factors associated with human activities also have a direct impact on the level of coal mine safety. Scholars should pay more attention to the possibility of triggering coal mine accidents and the extent of their impact after the coupling of various factors such as policy guidance, economic development, governmental actions, and miners’ living environment.

(2) Refine the study scale. Coal mine accident prevention and control strategies in China have regional characteristics [33], and studies in Chinese tend to focus on the national scale, while studies in English tend to focus on the status quo of individual provinces or single coal mines, which is not conducive to accurately grasping the regional differences of coal mine accidents in China. More attention should be paid to regional differences, and the spatial and temporal evolution characteristics and potential patterns of coal mine accidents in China should be explored according to coal-producing regions or governmental supervisory areas, which can provide more targeted references for practical work.

(3) Change the research thought. The integration of production–study–scientific research–practical application is the mainstream tendency currently. Taking into account the development status of China’s coal industry, simple research on method improvement or statistical analysis can no longer meet the demands of practical work, and it is urgent for scholars to provide new thoughts to enhance the transformation efficiency of achievements. Consequently, the improvement of the satisfaction of government and enterprise safety management needs by means of science and technology, and the improvement of real-time monitoring and risk warning capability of coal mine disasters during the production process, are the major directions at present.

## 6. Conclusions

(1) This paper presents an analysis of the literature on coal mine accidents in China from 1993 to 2022 by means of LSI + Apriori with the assistance of CiteSpace software and obtains the research history, research topics, cutting-edge features, and research discrepancies between Chinese and English in this field comprehensively and systematically. The analysis results can provide reference for related scholars to grasp the research progress in the field and complement the research shortcomings.

(2) The development of coal mine accidents as a research topic in China is consistent with the development status of China’s coal industry and basically follows the four stages of “statistical description, risk evaluation, mechanism research, and intelligent reasoning”. The development stages of studies about coal mine accidents in Chinese journals took place in the years of 1993–2001, 2002–2011, 2012–2016, and 2017–2022, and that of the English studies in 1993–1996, 1997–2011, 2012–2019, and 2020–2022.

(3) Chinese studies focus more on the analysis of accident characteristics and accident causative factors at large scale (nationwide) and mainly concentrate on four aspects: causative analysis, statistical analysis, monitoring and early warning, and safety management. The deep mining of massive accident information based on big data technology, the discovery and summarization of regional variations in safety levels, and risk monitoring and early warning based on new technologies are the popular directions of studies in Chinese. On the other hand, the English-language studies mainly focus on the injury to miners and the analysis of human causes, the studies are more concentrated on small-scale (single province or mine) or single-risk factor analysis, and the research topics are centered on occupational injury, causative analysis, and safety management, and they are starting to focus on the authenticity and public opinion guidance of news reports on coal mine accidents in China and the research on government safety strategies.

## Figures and Tables

**Table 1:** Topic and top term of accident characteristics statistics.

Topic	Subtopic	Silhouete *	Year	High-Frequency Terms
Roof accidents	#1 Roof accidents	0.915	1997	Roof accidents; Overburden movement; Roof caving; Coal face; Numerical simulation/Roof caving accident; Coal face; Prevention and control measures; Initial incoming pressure; Shock wave
Fire accidents	#13 Fire accidents	0.922	2000	Fire accident; Sound identification; Major and extraordinary accidents in coal mines; Gas and coal dust explosion; Coal and gas outburst/Mine safety; Fire accident; Cable fire; Flame-retardant cable; Mining cable
Water accidents	#5 Coal mine water inrush	0.822	2003	Water inrush accident; Hydrogeology; Coal mine floor; Coal mine water damage; Water prevention and control/Hydrogeology; Water exploration and discharge; Water damage prevention and control; Floor water inrush; North China coal mine
Gas accident	#2 Gas accidents	0.964	2008	Gas accident; Geological conditions; Gas accumulation; Gas concentration; Gas and coal dust accident/Digging surface; Gas in blind lane; Extraction fan; Transport pattern; Gas anomaly zone
Comprehensive analysis	#8 Safety evaluation	0.838	2010	Safety management; Safety production; Technical management; Safety policy; Accident prevention and control/Quantitative evaluation; Hierarchical analysis; Comprehensive evaluation; Rough set; Fuzzy comprehensive evaluation
	#0 Statistical analysis	0.856	2011	Statistical analysis; Prediction trend; Time series extrapolation analysis; Coal mine safety; Disaster avoidance system/Statistical analysis; Prevention and control countermeasures; Water damage; Research and judgment of probability; Water filling conditions

* Silhouette represents the homogeneity of cluster. Assuming that all clusters are the same size, the higher the Silhouette value, the stronger the homogeneity of the cluster. By contrast, if this value is too small, it means the degree of homogeneity parameter is meaningless.

**Table 2:** Topic and top term of accident causation.

Topics	Subtopic	Silhouete	Year	High-Frequency Terms
Causation analysis	#4 Comprehensive analysis	0.914	2008	Causation factors; Gas explosion; fault tree analysis; Fire accident; Grey correlation analysis/Minimum path set; Minimum cut set; Coal and gas outburst; Surface coal mine; Hazard source identification
	#3 Unsafe actions	0.736	2011	Unsafe action; 2-4 model; Behavioral causes; Coal mine gas explosion: Casualty laws/Gas explosion; Coupling laws; Accident characteristics; Accident prevention and control; HFACS
Knowledge graph	#18 Text clustering	0.918	2010	Text clustering; Ontology; Case matching; Information extraction; Knowledge discovery/Case inference; Emergency decision-making; Scenario retrieval; Gas accident; Pretrained language model
	#16 Knowledge graph	0.996	2021	Knowledge graph; Coal mine electromechanical equipment; neo4j; Smart Q&A; Risk management; Research hotspots; Research trends; Safety production; CiteSpace; Coal mine electromechanical equipment

**Table 3:** Topic and top term of coal mine safety management.

Topics	Subtopics	Silhouete	Year	High-Frequency Terms
Government-enterprise game	#7 Safety culture	0.847	2001	Lessons learned; Indicator control; Stress analysis; Essential safety; Safety costs/Coal mine enterprises; Safety culture; Personal behavior; Stress analysis; Accident cases
	#12 Illegal mining	0.966	2005	Illegal mining; Gas explosion; Disorderly management; Township coal mine; Individual coal kiln/Mine gas explosion; Individual coal kiln; Underground water inrush; Personal safety; Gas combustion
	#10 Safety supervision	0.933	2005	Regulatory system; Safety supervision; State-owned major coal mines; Safety production; Coal mine safety regulations/Fatality rate per 1 million ton of coal; Local coal mines; Coal prices; Industrial development; Fatal accidents
	#14 State functions	0.981	2006	Government supervision; Roof accident; Transportation accident; Accident investigation report; Liability accident/Local coal mines; Potential risks of accidents; Game model; Coal mine safety rules; End of investigation
Monitoring and early warning	#6 Risk evaluation	0.81	2007	Risk evaluation; Gas explosion; Hierarchical analysis method; Precontrol management; Unsafe behavior/Indicator system; Coal mine safety; Identification and evaluation; Fuzzy integrated evaluation method; Expert system
	#9 Digitalization	0.848	2012	Safety risk; b/s model; Early warning interval; Remote monitoring; Early warning system/Digitization; Database; Structural equation model; Case inference; Hazard source
	#11 Risk early warning	0.849	2012	Coal mine safety; Data mining; Risk warning; Influencing factors; Early warning system/Human factor analysis; System construction; Accident coding; Safety training; Risk early warning
Emergency rescue	#15 Emergency rescue	0.965	2017	Emergency rescue; Emergencies; Intuitionistic fuzzy set; Coal mine disaster accident; Weight adjustment/Case reasoning; Emergency supplies; Virtual drills; Emergency plans; Webgis

**Table 4:** Topic and top term of occupational injuries.

Topic	Subtopic	Silhouete	Year	Top Term
Descriptive statistics	#11 Injury statistics	0.997	1996	Human factor; Coal mine accident experience; Characteristics; 10-year tendency; Injury statistics/Human factor; Characteristics; China coal mine accident
	#5 Coal miner	0.893	2002	Coal miner; Physical job task; Living condition; Working condition; Individual characteristics/Underground coal mining injury; Working condition; Physical job task; Individual characteristics; Coal miner
Quantitative analysis	#14 Heat illness	0.931	2003	Heat illness; Mining industry; Measuring mining safety; Injury statistics; Work location/Blasting-related accident; Area security; Fly-rock phenomena; Heat illness; Work location
	#2 Occupational injury	0.749	2007	Case study; Spontaneous combustion; Gas explosion; Coal mining; Underground coal mine/Low temperature; Gas drainage; Gas concentration; Assessing operational risk; Coal mining enterprises

**Table 5:** Topic and top term of Coal Mine Disaster Mechanisms.

Topic	Subtopic	Silhouete	Year	Top Term
Accident analysis	#7 Water inrush	0.972	2009	Mechanized caving; Super great mining height; Strata pressure behavior; Abnormal gas emission; Thick main roof/Water inrush; Karst water; Mechanized caving
	#19 Flame	1	2009	Shock energy; Gas energy; Explosion flame; Flame speed; Flame duration
	#13 Coal and gas outburst	0.962	2011	Coal and gas outburst; Danger classification; Disasters control; Regional gas drainage; Greenhouse gas reductions/Disasters control; Regional gas drainage; Greenhouse gas reductions; In-seam directional long-holes; Gas drainage
	#12 Mine explosion	0.986	2011	Shock wave suppression; Gas explosion overpressure; Reflected pressure wave; Foam ceramics; Dust explosion/Particle size; Explosion Prevention; Dust explosion; Shock wave suppression; Gas explosion overpressure
	#0 Gas explosion	0.625	2013	Gas explosion; Gas hazards; High-gas tunnel; Strata behavior; Methane accumulation/Spontaneous combustion; Coal mining; Methane movement; Emergency evacuation; Emergency decision making
Comprehensive Analysis	#24 Correlation analysis	0.961	2009	Mine safety; Mine accidents; Correlation analysis; Association rules; Text message alerts
	#20 Accident recurrence	0.945	2017	Case-control study; Coal mine rock damage; Methane ignition mechanism; Postexplosion observation; Laboratory coal dust

**Table 6:** Topic and top term of Safety Management.

Topic	Subtopic	Silhouete	Year	Top Term
Coal safety	#16 Safety culture	0.873	2003	Safety culture; Accident causation; Awareness; Mine rules; Coal miners’ safety behavior
	#1 Mine safety	0.949	2013	Mine safety; Coal mining; Accident causes; Contingency tables; Multiplayer game/Safety climate; Safety management; Safety performance; Disaster control; Environmental impacts
	#25 Training	1	2015	Occupational safety and health; Accident prevention; Training; Virtual reality; Underground coal miners
Risk assessment	#4 Coal mining	0.94	2005	Coal mining; Reporting bias; Establishment employment size; Injury reporting; Denominator-free methods; Lost time measures; Job safety; Statutory days; Incidence rates
	#3 Risk assessment	0.959	2012	Risk assessment; Gas explosion accidents; Unsafe behaviour; Risk function; Explosion risk/Industrial accident; Connectionism strategy; Coal mine accident; Emergency management; Parallel constraint satisfaction
	#15 Supervision	0.952	2015	Accident precursor; Supervision; Accident coverup; Disaster prevention ability; Effect

## Data Availability

Not applicable.
